# A Retrospective Assessment of Occupational Exposure to Elemental Carbon in the U.S. Trucking Industry

**DOI:** 10.1289/ehp.1002981

**Published:** 2011-03-29

**Authors:** Mary E. Davis, Jaime E. Hart, Francine Laden, Eric Garshick, Thomas J. Smith

**Affiliations:** 1 Department of Urban and Environmental Policy and Planning, Tufts University, Medford, Massachusetts, USA; 2 Exposure, Epidemiology and Risk Program, Department of Environmental Health, Harvard School of Public Health, Boston, Massachusetts, USA; 3 Channing Laboratory, Brigham and Women’s Hospital and Harvard Medical School, Boston, Massachusetts, USA; 4 Department of Epidemiology, Harvard School of Public Health, Boston, Massachusetts, USA; 5 Pulmonary and Critical Care Medicine Section, Medical Service, VA Boston Healthcare System, West Roxbury, Massachusetts, USA

**Keywords:** air pollution, diesel, lung cancer, occupational health, traffic exposure, trucking industry

## Abstract

Background: Despite considerable epidemiologic evidence about the health effects of chronic exposure to vehicle exhaust, efforts at defining the extent of risk have been limited by the lack of historical exposure measurements suitable for use in epidemiologic studies and for risk assessment.

Objectives: We sought to reconstruct exposure to elemental carbon (EC), a marker of diesel and other vehicle exhaust exposure, in a large national cohort of U.S. trucking industry workers.

Methods: We identified the predictors of measured exposures based on a statistical model and used this information to extrapolate exposures across the cohort nationally. These estimates were adjusted for changes in work-related conditions over time based on a previous exposure assessment of this industry, and for changes in background levels based on a trend analysis of historical air pollution data, to derive monthly estimates of EC exposure for each job and trucking terminal combination between 1971 and 2000.

Results: Occupational exposure to EC declined substantially over time, and we found significant variability in estimated exposures both within and across job groups, trucking terminals, and regions of the United States. Average estimated EC exposures during a typical work shift ranged from < 1 μg/m^3^ in the lowest exposed category in the 1990s to > 40 μg/m^3^ for workers in the highest exposed jobs in the 1970s.

Conclusions: Our results provide a framework for understanding changes over time in exposure to EC in the U.S. trucking industry. Our assessment should minimize exposure misclassification by capturing variation among terminals and across U.S. regions, and changes over time.

Although there is considerable concern about the health effects of chronic exposure to vehicle exhaust, efforts to define the extent of risk have been limited by the lack of historical exposure measurements suitable for use in epidemiologic studies and for risk assessment. There is no standard accepted practice to retrospectively estimate exposure levels. Despite significant progress and research on this topic in the 1990s, historical exposure reconstruction remains a demanding and time-consuming process (Ward et al. 2003) that is driven primarily by the available data (Davies et al. 2009; Stewart 1999), which are often incomplete or missing for important historical exposure periods (Seixas and Checkoway 1995). However, without an accurate and historically relevant exposure model, it is difficult to obtain unbiased estimates of lifetime dose, resulting in exposure misclassification and ultimately risk estimates that are likely to be biased toward the null.

In previous epidemiologic studies of U.S. railroad workers exposed to diesel exhaust (Garshick et al. 2004; Laden et al. 2006), estimates of historical exposures were not available for quantitative risk estimates (Woskie et al. 1988a, 1988b). Studies of trucking industry workers reported associations between lung cancer and estimated cumulative exposure to diesel exhaust based on vehicle miles traveled by heavy-duty trucks, historic truck emission factors, and fuel consumption (Bailey et al. 2003; Steenland et al. 1998). These studies made linear extrapolations of historic exposure (spanning ≥ 20–30 years) assuming that the estimated exposures applied uniformly across a spatially dispersed cohort. This approach is limited in its ability to address temporal trends and ignores the impact of spatial variability altogether. A more recent retrospective assessment of exposure to diesel exhaust in U.S. miners used historical measurements of carbon monoxide (CO) along with information on engine horsepower and mine ventilation to reconstruct exposure to elemental carbon (EC) (Vermeulen et al. 2010a). However, the correlation between current CO and EC measurements in this study was modest (*r* = 0.4; Vermeulen et al. 2010b). The lack of available exposure data is not unique to the miner’s study, but instead highlights the difficultly of obtaining reliable historically relevant exposure data.

In this article, we provide a detailed description of the methods used to retrospectively estimate exposure to EC in particulate matter (PM) ≤ 1 μm in aerodynamic diameter (PM_1_) in an occupational cohort of U.S. trucking industry workers. We designed the approach to exposure assessment based on specific job duties and work locations, and unlike previous investigators, we identify the predictors of exposure in a statistical model. More specifically, we used the baseline exposure model developed from our industrial hygiene assessment of the industry to predict unmeasured on-site worker exposures for the study cohort as a whole in 2000. We then extrapolated these estimates to previous years, adjusting for changes in air pollution background and work-related exposure conditions over time, to derive monthly EC estimates from 1971 to 2000 for each job and terminal location.

## Materials and Methods

*Trucking Industry Particle Study.* The Trucking Industry Particle Study (TrIPS) was designed as a joint effort in exposure assessment and epidemiology (Garshick et al. 2002) in cooperation with the International Brotherhood of Teamsters and four large U.S. trucking companies. As part of this study, we performed a comprehensive exposure assessment of the U.S. trucking industry in which > 4,000 environmental samples were collected between 2001 and 2006. During the study period, we visited 36 different trucking terminals that were randomly selected to be regionally representative of the full set of 139 large terminals in operation in 2000 (Smith et al. 2006). For a map of the sampled trucking terminal locations, see Supplemental Material, Figure 1 (doi:10.1289/ehp.1002981).

**Figure 1:**
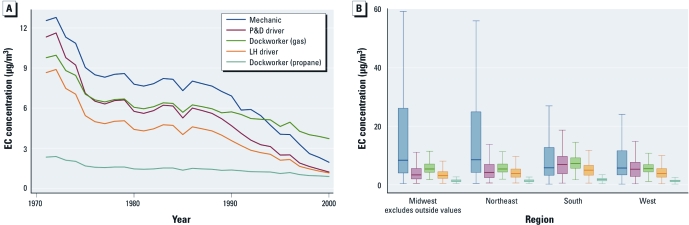
(*A*) Median annual EC trends across select job categories. (*B*) EC trends across select job categories by census region. The boxes indicate 25th, 50th (median), and 75th percentiles. Extreme outliers are excluded for display purposes, and the length of the whiskers are bounded from above by 1.5 times the interquartile range.

This large-scale exposure assessment was conducted in combination with a retrospective epidemiologic cohort study of lung cancer mortality among 55,000 trucking industry workers who were employed in 1985 at these four trucking companies with mortality follow-up through 2000. We obtained detailed work history records to link the subjects to exposures that included a chronological series of work locations, job titles, and years worked in each job. For a description of the various jobs in this occupational cohort, see Supplemental Material, Table 1 (doi:10.1289/ehp.1002981).

**Table 1:** Historical occupational EC in the trucking industry (μg/m^3^).

Table 1. Historical occupational EC in the trucking industry (μg/m^3^).																
								Zaebst study*a* (1988–1989)								
		TrIPS*b* (2001–2006)						Measured						Predicted*c *GM		Historical*d* multiplier
Job		No. of observations		GM		GSD		No. of observations		GM		GSD				
Background/clerks		699		0.5		3.2		23		1.1		2.0		1.1		NA*e*
LH		388		1.1		2.3		72		3.8		2.3		2.3		1.7
P&D (warm)		535		1.2		2.3		25		6.3		1.9		2.5		2.5
P&D (cold)		197		1.0		3.5		31		2.8		1.6		2.2		1.3
Dockworker (propane)		632		0.9		2.2		12		1.3		2.0		1.36		1.0
Dockworker (diesel)								54		27.2		1.7				20.9*f*
Dockworker (gasoline)								9		5.5		1.6				4.2*f*
Mechanics (warm)		96		1.5		2.4		38		4.8		2.4		2.0		2.4
Mechanics (cold)		53		4.3		4.0		42		28.0		2.8		7.6		3.7
Abbreviations: GM, geometric mean; GSD, geometric standard deviation; NA, not applicable. **a**Zaebst et al. (1991). **b**Smith et al. (2006) and Davis et al. (2009). **c**Represents predictions from baseline exposure model adjusted for background conditions. **d**Historical multipliers were generated based on relationship between measured geometric means from Zaebst et al. (1991) and those predicted by baseline exposure model, unless otherwise specified; linear extrapolation applied to years between study periods. **e**Year-specific background multipliers applied for 1971–1999 are based on archived COH data. **f**Historical multipliers for dockworkers driving diesel or gasoline forklifts were developed based on ratio to propane as observed by Zaebst et al. (1991). Empty cells indicate that no diesel or gasoline forklifts were observed for Smith et al. 2006.																

The exposure monitoring plan is described in detail elsewhere (Davis et al. 2006; Smith et al. 2006). Briefly, concentrations of vehicle exhaust markers (EC and organic carbon) in PM_1_ and in PM ≤ 2.5μm in aerodynamic diameter (PM_2.5_) were simultaneously measured for individual workers, indoor work areas, truck cabs, and ambient background conditions at the 36 trucking terminals. EC levels were determined using the NIOSH (National Institute for Occupational Safety and Health 1998) 5040 method, whereby PM_1_ was collected on a 22-mm quartz tissue filter, preceded by a precision machined cyclone separator (SCC1.062 Triplex; BGI, Inc., Waltham, MA) to remove particles > 1 μm in aerodynamic diameter. Because we were interested in vehicle exhaust from diesel-fueled sources, EC in PM_1_ was chosen as our primary marker of vehicle exhaust (Fraser et al. 2003; Kleeman et al 2000; Riddle et al. 2008; Schauer 2003; Sheesley et al. 2009). A more detailed discussion of the choice of EC as a surrogate marker of diesel exhaust is provided in the Supplemental Material (doi:10.1289/ehp.1002981).

**Figure 2:**
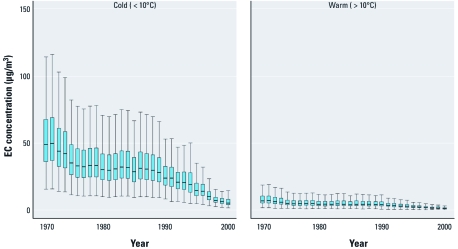
Annual EC trends in mechanics by temperature profile. For display purposes, the *y*-axis is scaled to exclude extreme outliers, which are defined as values > 75th percentile multiplied by the interquartile range. The boxes indicate 25th, 50th (median), and 75th percentiles. Extreme outliers are excluded for display purposes, and the length of the whiskers are bounded from above by 1.5 times the interquartile range.

*Baseline exposure model.* We used structural equation modeling (SEM) techniques (Sanchez et al. 2005) to predict shift-specific personal EC levels for the on-site terminal workers, including dockworkers (who load and unload cargo), mechanics, clerks, and hostlers (on-site drivers that move trailers using small specialized tractor units). In brief, personal job-specific exposures were predicted by smoking status and work area exposures (*R*^2^ = 0.64); work area exposures were predicted by terminal-specific characteristics, ventilation, job location in the terminal, and matching background exposures from the area around the terminals (*R*^2^ = 0.64); and background exposures were predicted by local weather characteristics, proximity to a major road, industrial land use characteristics around the terminal, and regional location within the United States (*R*^2^ = 0.51) (Davis et al. 2006) [for additional details on the structure of the exposure model, see Supplemental Material, Table 2, Figure 2 (doi:10.1289/ehp.1002981)]. The modeling approach was validated using additional exposure data collected during a series of six repeat site visits conducted after the initial 36 terminal sampling trips (Davis et al. 2009).

**Table 2:** Summary statistics of shift-level EC predictions by job per decade (μg/m^3^).

Table 2. Summary statistics of shift-level EC predictions by job per decade (μg/m^3^).																		
		1971–1980						1981–1990						1991–2000				
Job group		Mean		Median		SD		Mean		Median		SD		Mean		Median		SD
Background/clerks		1.79		1.65		0.74		1.25		1.20		0.40		0.80		0.75		0.31
Dockworkers*a *(diesel)		40.80		37.25		17.28		32.06		29.86		12.19		24.73		22.83		9.96
Dockworkers*a *(gasoline)		8.20		7.49		3.47		6.44		6.00		2.45		4.97		2.00		4.59
Dockworkers*a *(propane)		1.95		1.78		0.83		1.53		1.43		0.58		1.18		1.09		0.48
Mechanics (all)		19.66		9.72		22.48		15.23		7.66		16.98		7.64		3.86		9.91
Mechanics (warm climate)		7.75		6.33		5.05		6.08		5.07		3.75		3.16		2.56		2.33
Mechanics (cold climate)		43.23		37.72		24.86		33.57		29.77		18.27		16.43		13.19		12.79
LH drivers/hostlers		6.40		5.88		2.64		4.46		4.26		1.45		2.21		2.01		1.04
P&D drivers (warm)		10.41		9.59		4.16		7.23		6.97		2.25		3.09		2.77		1.64
P&D drivers (cold)		4.56		4.15		1.99		3.18		2.95		1.12		1.79		1.64		0.80
**a**Dockworker exposure predictions not relevant to all time periods; based on company reported fuel-use profiles.																		

Separate exposure models were constructed for drivers who worked off-site delivering and picking up freight (Davis et al. 2007), including local pickup-and-delivery (P&D) drivers and long-haul (LH) drivers whose exposures could not be modeled explicitly within the SEM because of the dynamic nature of their exposures. Driver EC exposures were moderately correlated with background EC levels measured at their home terminals, with stronger correlations for local P&D drivers (*r* = 0.4–0.5; *p* < 0.01) than for LH drivers (*r* = 0.2–0.4; *p* < 0.01). Measured EC levels inside the driver cabs were also significantly higher when the windows were predicted to be open versus shut [*p* < 0.05; for further detail on window status, see Supplemental Material (doi:10.1289/ehp.1002981)]. Differences in EC measurements across driver groups (P&D vs. LH) and by driver smoking status were not statistically significant.

*Spatial extrapolation approach.* For the present study, we used the SEM exposure model and characterization of driver exposures described above (Davis et al. 2007) to extrapolate EC exposures to workers at the 103 additional large terminals in the epidemiologic cohort that were not part of the original exposure assessment. The SEM used for this purpose did not include smoking or relative humidity because these data were not available for the entire cohort of workers and terminals. However, excluding these variables did not appreciably affect model estimates. Using input data from each of the terminals and the estimated coefficients from the modified SEM, we extrapolated exposures spatially across the entire cohort for the year 2000 and calculated a monthly exposure estimate for each job and terminal combination. The year 2000 was chosen as the base year for the exposure extrapolation efforts because it represents the final year of follow-up in our epidemiologic cohort.

To extrapolate driver exposures spatially across the cohort, we constructed scaling factors that related measured driver EC to model-based background predictions. Specifically, we determined ratios of median driver to terminal background EC for each driver type. This ratio resulted in a multiplier of 2.1 for LH drivers and hostlers, indicating that exposures for these drivers were typically 2.1 times higher than terminal background conditions. For P&D drivers, we calculated separate multipliers for warm- and cold-weather conditions to account for the impact of open cab windows in the truck cabs that were not air conditioned. The multiplier for P&D driver exposures to background levels was 2.3 (window open) in warm-weather conditions (> 10°C) and 2.0 for colder temperatures (window shut).

*Temporal extrapolation approach.* To account for changes in job-related exposure characteristics over time, we conducted a comprehensive historical review of work practices in the industry, including the introduction of diesel-fueled vehicles across job groups and companies [see Supplemental Material, Table 3 (doi:10.1289/ehp.1002981)]. Although LH drivers began operating diesel-fueled trucks in the 1950s, the P&D fleet was predominantly gasoline-powered until the 1970s and 1980s. Such changes may not have affected drivers directly but would have affected the composition of highway and road emissions, which increased EC exposures as the whole industry changed truck engine types over a short period. Diesel forklifts were in operation on the terminal docks for only a relatively brief period of time during the 1980s at three of the four companies; otherwise, gasoline- or propane-powered forklifts were used. Propane-powered forklifts predominated after diesel forklifts were phased out in the early 1990s.

We derived job-specific historical multipliers to extrapolate baseline exposure model estimates derived for 2000 to earlier periods based on EC exposure data from a study of unionized trucking industry workers conducted in the late 1980s (Zaebst et al. 1991). Specifically, we used the structure of our exposure model along with historical input data on the model covariates to predict job-specific EC exposures for workers in the earlier study; we used the ratio of our model-based predictions to the measured values reported by Zaebst et al. (1991) to adjust for changes in work-related conditions over time. In addition, because forklifts used during the TrIPS exposure assessment were powered by propane fuel only, we used data from Zaebst et al. (1991) to develop fuel use multipliers by comparing EC concentrations from propane with concentrations related to diesel- and gasoline-powered forklifts.

There is no widespread national database for ambient EC or PM_1_ (either current or historical) that could be used to adjust for the indirect effect of changes in ambient EC levels over time on work-related EC exposures across the cohort. For this reason, we explored trends in a surrogate EC marker known as the coefficient of haze (COH). COH represents an early air pollution monitoring technique developed in 1953 and widely employed in the 1960s and 1970s (U.S. Environmental Protection Agency 1983). The COH measurement method has been shown to be strongly predictive of EC (*R*^2^ = 0.94) (Cass et al. 1983), and COH has recently been used to characterize changes in diesel-related PM exposure conditions over time (Davis et al. 2010; Kirchstetter et al. 2008). Although COH measurements away from traffic reflect a variety of air pollution sources, diesel dominates COH levels observed near highways and roads.

Most historical COH data have been lost because of changes in data storage methods. However, we obtained monthly COH averages for 26 separate New Jersey locations for 1971–2000, and hourly COH data from California for 1980–2000. In a separate analysis, we constructed nationwide models of annual exposure to PM_10_ and nitrogen dioxide for 1985–2000 (Hart et al. 2009). We decided to use the New Jersey COH data to estimate background EC conditions over time in our cohort because these data provided the longest unbroken panel to reconstruct historical exposures and because they corresponded to the available data on background EC conditions during the Zaebst et al. (1991) assessment. A more detailed description of the choice of New Jersey COH and comparison with the other exposure data is provided in the Supplemental Material (doi:10.1289/ehp.1002981). To explicitly incorporate a background trend based on COH into the exposure model, we derived ratios comparing the median annual COH value in each year with the estimate for base year 2000 and used them to adjust annual background EC predictions for 1971–1999.

In summary, we first used the baseline exposure model developed from our TrIPS industrial hygiene assessment of the industry to predict unmeasured on-site worker exposures for the study cohort as a whole in 2000. Next, we extrapolated these estimates to previous years. To derive monthly EC estimates from 1971 to 2000 for each job and terminal location, we adjusted for changes in air pollution background and work-related exposure conditions over time.

## Results

Table 1 provides data from the TrIPS (Smith et al. 2006) and Zaebst et al. (1991) exposure assessments, along with the job-specific historical multipliers that we developed from this comparison and applied to our cohort. Not surprisingly, estimated exposures were significantly higher for 1988–1989 than for 2001–2006 for all job groups. For dockworkers driving propane forklifts, the geometric mean of the predicted EC value was approximately equal to the geometric mean reported by Zaebst et al. (1991) (predicted, 1.36; actual, 1.30) after accounting for elevated background conditions in the past. However, a multiplier to account for the type of fuel used in forklifts driven by these workers indicated the impact of higher emitting fuels, namely, diesel and gasoline, on past exposures.

In our cohort, mechanics experienced the greatest change in duties over time of all the jobs. Previously, mechanics in the trucking industry performed most major repair and replacement work, whereas currently they perform only minor repairs and preventive maintenance. Even after accounting for elevated background conditions, past mechanic exposure levels were still 2.4–3.7 times higher than were the levels predicted for warm- and cold-weather conditions, respectively, with higher exposures during cold weather when repair bay doors are closed (Table 1) (Smith et al. 2006).

Driver exposures have also changed over time because of alterations in work practices. We estimated driver exposures to be between 1.3 and 2.5 times greater during the late 1980s than during the early 2000s, even after controlling for higher background levels in the past (Table 1). The estimated impact of window status (at 10°C cutoff) for P&D drivers was greater in the past, which is likely due to the different mix of traffic sources present and the greater intensity of these sources. We found no sizable window effect in LH drivers, whose truck cabs were equipped with air conditioning throughout the entire study period.

Table 2 provides a numerical summary of predicted shift-level EC exposures by job group over time. Clerk EC exposures were indistinguishable from background levels, because no immediate exposure source is present in these indoor work locations. As such, exposure levels for these workers are assumed to be equivalent to background conditions throughout our study. Also, the use of diesel forklifts on terminal docks varied by company, and the summarized occupational exposures in Table 2 are not relevant to all periods; that is, the diesel dockworker phase is limited primarily to the 1980s. This assumption also applies to dockworkers exposed to gasoline-powered forklifts, because propane-powered forklifts dominated after the 1990s. Although we present the full panel of exposure estimates for each job category here, cumulative exposure estimates for the epidemiologic model are based on individual work histories and on the profile of fuel use that was provided by the participating companies.

The EC levels for mechanics were significantly higher than those for both driver groups in each decade noted in Table 2 and were higher than for propane dockworkers (*p* < 0.01). These results are consistent with the measured exposure data observed by both Smith et al. (2006) and Zaebst et al. (1991). EC exposures were also higher among mechanics than among gas dockworkers during the relevant exposure periods (pre-1990s; *p* < 0.01). Exposure conditions were similar between diesel dockworkers and cold-weather mechanics during the relevant period of diesel forklift use (before 1990).

Figure 1A displays median annual trends in estimated EC exposure levels across select job groups. Because the EC estimates for diesel dockworkers are nearly four times higher than those for the next highest exposed job category (mechanics), we excluded this group from Figure 1A to better illuminate trends in the other job categories. However, it is clear that the use of diesel forklifts on the semienclosed docks contributed greatly to exposure levels of dockworkers during that time period. Estimated EC exposures in the trucking industry trend higher in the past for all job groups, with the lowest levels observed during the base year 2000. These temporal trends in the exposure profile across job categories reflect both changes in work practices and elevated background conditions over time. All annual exposure estimates differed significantly across job categories (*p* < 0.05), with the exception of exposures among gas dockworkers and P&D drivers, which were not significantly different in 1975 and 1979. When we compared driver groups, exposure estimates were similar during more recent periods, although these small differences were still statistically significant. The driver differences widen over time as P&D exposure levels increase more rapidly than do LH exposures in the past. This increase is due to the larger effect of background pollution on P&D truck cabs in warm-weather conditions (open windows), which was also higher in the past.

Figure 1B provides evidence of regional differences both within and between the various job groups across U.S. Census regions. Mechanic exposures were significantly higher in the Midwest and Northeast (*p* < 0.01), whereas exposures in the other job groups were comparatively higher in the South and West (*p* < 0.01).

Figure 2 illustrates the importance of temperature on mechanic exposure trends over time. Estimated mechanic exposures were significantly lower (*p* < 0.01) in warm weather than in cold weather. This result is due to the large impact of open-door ventilation in the semienclosed work environment of the mechanic shop and is consistent with measured exposure data from both studies (Smith et al. 2006; Zaebst et al. 1991).

## Discussion

This article outlines the approach we used to estimate occupational exposure to EC, a surrogate marker of vehicle exhaust with a significant contribution of diesel exhaust particles, in the U.S. trucking industry between 1971 and 2000. We used SEM techniques to construct a quantitative model of job- and terminal-specific exposures across the United States in 2000 (Davis et al. 2006). We then retrospectively applied these estimates after adjusting for historic changes in work practices and background conditions to generate monthly EC estimates for each job and terminal location from 1971 to 2000. Our approach was substantially different from previous efforts because we combined an extensive assessment of current exposure with historical data in a statistical model to extrapolate exposures spatially and temporally across the cohort.

We observed evidence of a number of important trends across job groups. By far the largest job-related exposure impact was the past use of diesel-powered forklifts on terminal docks, and we identified forklift engine type as a major source of variability within the dockworker job group. Diesel-fueled forklifts provided the largest contribution to dockworker EC exposures, followed by gasoline-fueled forklifts, whereas work-related exposure from propane-powered forklifts was very low.

The results also show significant variability in exposures among mechanics. Although mechanic exposures at rural terminals in warmer climates were similar to regional background levels, workers in the same job category working at cold-climate urban terminals experienced the highest exposure levels in the entire cohort because of reduced ventilation when terminal bay doors are kept closed. In addition, mechanic exposures in warmer climates were relatively stable within a given year, whereas mechanics working in colder climates experienced wide seasonal exposure shifts, resulting in wider ranges of predicted values for mechanics in the Midwest and Northeast compared with mechanics in the South and West.

In contrast, exposures for P&D drivers, which come from outside of the truck cab, are increased during warmer temperatures and in warmer climates because these trucks are not equipped with air conditioning and cab windows are more likely to be open when it is warm outside. This effect was not as evident for LH drivers, whose truck cabs were equipped with air conditioning throughout the study period. Because of the greater contribution of background conditions to exposures in P&D drivers, P&D exposure levels were higher than LH exposures in the past, consistent with higher background exposures.

Overall, both geographic and temporal trends, which have not been evaluated in previous studies, were important exposure modifiers for workers in this occupational cohort. In addition, we identified significant variability in exposures within job groups across terminal locations. These results highlight the need in this and similar occupational health studies to control for these important sources of variability when estimating cumulative exposures. Even relatively minor estimation errors can result in substantial misclassification bias (Stayner et al. 1999; Stewart 1999), ultimately limiting the power to detect exposure–disease relationships (Ahrens and Stewart 2003). Unfortunately, historical exposure reconstruction efforts are often hampered by incomplete exposure information, and failure to account for temporal changes when estimating exposures can introduce a significant amount of error in chronic epidemiologic studies (Seixas and Checkoway 1995; Ward et al. 2003).

Past studies of diesel exhaust have generally lacked the historically relevant data necessary to identify many of these important exposure trends (Stayner et al. 1998) and have often lacked the work records necessary to identify job-related exposure differences and patterns over time (Bunn et al. 2004; Hesterberg et al. 2006; Valberg and Watson 2000). In our previous study of diesel exposure in U.S. railroad workers (Woskie et al. 1988a, 1988b) we applied a single national average of total suspended particulates to control for the contribution of background levels. In an earlier study of the U.S. trucking industry, Steenland et al. (1998) adjusted job-related diesel exposures historically using information on vehicle miles traveled, fuel consumption, and estimated emission factors of diesel engines. However, the relationship between occupational exposures and these characteristics was untested in the original exposure model proposed by Steenland et al. (1998), so there is no way to justify the use of these factors as temporal control factors. A follow-up study that was conducted by Bailey et al. (2003) applied simulation methods to better incorporate the impact of these temporal controls and evaluate model uncertainty.

A more recent assessment of diesel exposure in U.S. miners used CO levels to identify trends in work-related EC exposure conditions over time because of the absence of historical EC data for this cohort (Vermeulen et al. 2010a). However, the correlation between CO and EC in the study locations where the miners worked was only moderate (Vermeulen et al. 2010b), and the use of CO as a surrogate for EC may lead to exposure misclassification bias. In the present study, historical EC data were available from the Zaebst et al. (1991) industrial hygiene survey of the trucking industry, which we used to explicitly adjust for changing EC exposure levels over time in our cohort. Also, the COH data we used to control for the indirect effect of changing background EC conditions over time at the terminals provides a much stronger surrogate marker of EC than does CO (Cass et al. 1983; Vermeulen et al. 2010b).

*Limitations.* The retrospective assessment of exposures in this cohort required many assumptions that introduced uncertainty in the exposure estimates across workers and terminal locations in the cohort. The use of COH data from a single state (New Jersey) to estimate background exposure trends for the rest of the United States is problematic, because the temporal trend may vary across different locations. However, we captured spatial differences across terminal locations within the exposure modeling structure, and therefore the major limiting assumption of using the New Jersey COH data is that these spatial differences remain constant over time.

Another limitation is that the present analysis addresses the historical extrapolation of worker exposures only back to 1971, which is the earliest year for which reliable exposure estimates could be constructed. Although this extrapolation period does not cover the entire period of exposure relevant to the epidemiologic cohort, the missing years before 1971 represent a small percentage of the person years (8%) in the epidemiologic cohort. We are currently exploring options for assigning exposure levels for periods before 1971.

In addition, exposure estimates for specific workers depend in part on the accuracy of fuel-use timelines provided by the trucking companies. We have attempted to limit exposure misclassification by validating the exposure model for the current period as well as controlling for changes over time using exposure data from Zaebst et al. (1991). In the upcoming epidemiologic analyses of this cohort, we are working to construct a range of exposure values to incorporate uncertainty around the point estimates presented here. At present, there is no established method for incorporating exposure uncertainty into a Cox proportional hazards model.

## Conclusion

Our historical exposure model represents a significant improvement over exposure estimates used in other occupational studies of diesel and vehicular exhaust. Although the benefits of statistical modeling as a retrospective assessment tool have been noted in the past (Seixas and Checkoway 1995), the use of SEMs described here provides a novel approach that represents both a more realistic and replicable framework for estimating cumulative exposure to EC in our national cohort. Overall, our assessment should reduce exposure misclassification by accounting for variation among terminals and U.S. regions, as well as changes over time.

## Supplemental Material

(116 KB) PDF
