# Supplementary material for: A Retrospective Assessment of Occupational Exposure to Elemental Carbon in the U.S. Trucking Industry
Source: Environ Health Perspect. 2011 Mar 29;119(7):997–1002. doi: 10.1289/ehp.1002981 (PMC3222985; doi:10.1289/ehp.1002981)
Supplement: (116 KB) PDF [file ehp.1002981.s001.pdf]

Supplemental Material

A Retrospective Assessment of Occupational Exposure to Elemental Carbon in the US Trucking Industry

Davis ME, Hart JE, Laden F, Garshick E and TJ Smith

Table of Contents **Page**

Sampling Locations S-2
Figure 1: Map of TrIPS Trucking Terminals

TrIPS Job Descriptions S-2
Table 1: Job Descriptions in the Trucking Industry Cohort

Choice of EC as Diesel Exhaust Exposure Marker S-3

Structure of Baseline Exposure Model S-4
Figure 2: EC SEM Pathway Diagram and Results
Table 2: Variables in SEM Exposure Model

Window Status Description S-5

Diesel Fuel Timeline S-5
Table 3: History of Diesel Use in the Four TrIPS Companies

Selection of EC Background Trend S-6
Figure 3: Comparison of Background Trends 1985-2000

References S-7

Sampling Locations

Figure 1: Map of TrIPS Trucking Terminals

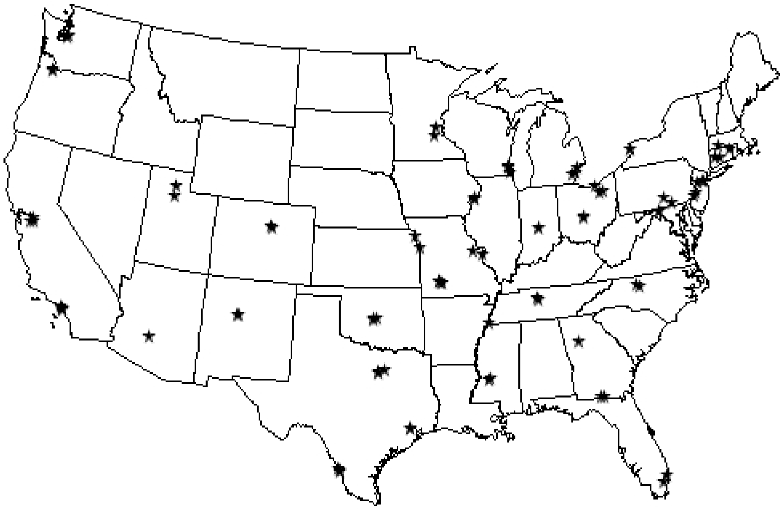

TrIPS Job Descriptions

Table 1: Job Descriptions in the Trucking Industry Cohort

Job Title	Job Description
**Terminal Workers**	
Dockworker	Works onsite in loading dock and loads/unloads cargo; may operate forklifts
Mechanic	Works onsite in truck repair shop and performs tractor maintenance; job may include fueling
Clerk	Works onsite in terminal offices and include cashiers, dispatchers, customer service representatives, and others not regularly near diesel vehicles
**Drivers**	
Hostler	Works onsite driving small specialized tractor units that move trailers around the terminal
Pickup and Delivery (P&D) Driver	Drives tractors or smaller single-bodied trucks within cities and rural areas; picks up and delivers cargo between terminal and consumer; truck cabs are not equipped with air conditioning
Long Haul (LH) Driver	Drives heavy duty tractor-trailer trucks long distances between cities; truck cabs are equipped with air conditioning

Choice of EC as Diesel Exhaust Exposure Marker

There is considerable concern about the health effects of chronic exposure to vehicle exhaust that have been associated with cancer and heart disease. However, the complexity of exhaust components – a mixture of gases, e.g. CO, SO_2 and NO_2, organic vapors and droplets, and submicron carbon particles with adsorbed toxic components and differences across different vehicle sources and operating conditions – have limited efforts at defining exposures and their contributions to risk. The EC particles with their adsorbed and condensed organic components are one of the main suspects for toxic activity by $PM_{2.5}$.

Exhaust particulate from diesel vehicles in the time periods of interest include an EC core with OC compounds on its surface. Although exhaust particles from spark-ignition vehicles include EC, they include greater amounts of OC (Kleeman et al. 2000). Source apportionment studies that we conducted in trucks and terminals indicate that most of the EC is from diesel sources (Sheesley et al. 2008; Sheesley et al. 2009). Although other non-vehicular combustion sources may contribute to EC and the proportion of EC emitted by diesel engines may vary based on operating conditions, our findings are consistent with previous source apportionment studies in the US (as reviewed in Schauer 2003) and with previous engine emissions and roadway studies (Kleeman et al 2000; Fraser et al. 2003; Riddle et al. 2008). Furthermore, as our EC data were collected in and around trucking terminals and in truck cabs, it is likely that only vehicular sources meaningfully contributed to EC.

Structure of Baseline Exposure Model

Figure 2 represents a pathway diagram of the SEM used to estimate EC, while Table 1 provides a description of the covariates in the model. In the diagram, the numbers inside the boxes represent the R^2 and equation-specific constants, while the numbers adjacent to the arrows represent the covariate coefficients.

Figure 2: EC SEM Pathway Diagram and Results

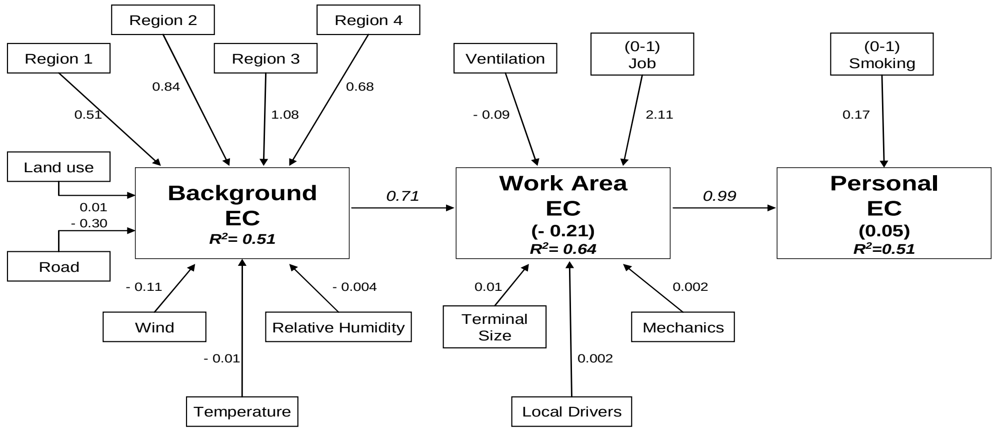

Table 2: Variables in SEM Exposure Model

	Equation 1	Equation 2	Equation 3
**Endogenous Variables**	Personal EC	Work Area EC	Background EC
**Exogenous Variables**	Smoking	Job: 0=dockworker, 1=mechanic	Relative Humidity<br>Temperature (C^0)<br>Wind: Windspeed (km/h)
	Work Area EC	Terminal Size: acreage<br>Mechanics: # employed<br>Local Drivers: # employed	Road: distance from interstate<br>0 if <500m, 1 if >500m
		Ventilation: Temperature*job	Landuse: % land designated Industrial, Commercial, Transportation within 100m radius of terminal
		Background EC	Region 1: Midwest (0/1)<br>Region 2: Northeast (0/1)<br>Region 3: South (0/1)<br>Region 4: West (0/1)

Window Status Description

We were able to approximate the window status of sampled trucks using a combination of in-cab CO_2 levels and the temperature differential inside-outside the truck cabs. Based on these data, we established a temperature cutoff of 10^0 Celsius as an indicator of open/shut windows in non-air conditioned truck cabs. The window predictions provided evidence of a significant external source of EC, with higher concentrations when the windows are assumed open ($p<0.01$) than when they were predicted to be shut. As further evidence of this 'window effect,' the relationship only held for drivers in truck cabs without air conditioning (P&D) and not for others where the in-cab temperatures could be regulated with air conditioning (LH).

Diesel Fuel Timeline

Table 3: History of Diesel Use in the Four TrIPS Companies

		Date			
Equipment	Diesel Use	Company 1[a]	Company 2	Company 3	Company 4
Long-haul trucks	First Used	1957	1952	1951	1955
	100%[b]	1962	1954	1961	1965
P&D trucks	First Used	1978	1977	1974	1972
	100%	1987	1992	1980	1983
Forklifts	Introduction	None used	1979	1986	1982
	100%		1986	1986	1984
	Phase-out		1990	1991	1992
	Last Used		1996	1994	1996

[a] To maintain company confidentiality, the companies are referred to as 1 through 4
[b] Date by which all of the vehicles in the fleet were diesel

Selection of EC Background Trend

To verify that the NJ data represented a reasonable trend in ambient EC conditions over time at the terminals in the cohort, we compared the slope of the median annual COH levels in NJ to a series of vehicle exhaust markers available over a more limited time period. This included the median annual averages of COH from CA (available 1980-2000), as well as the median annual values generated from a spatial mapping of PM_{10} and NO_2 (available 1985-2000). For a detailed description of the PM_{10} and NO_2 national exposure maps, see Hart et al. (2009).

Figure 2 provides a graphical comparison of these data during the period for which all are available. The downward trend in COH (both NJ and CA) is much steeper over time than it is for PM_{10} or NO_2. Since COH is more closely related to EC than either PM_{10} or NO_2, and these datasets also provided the most limited time series, they were ruled out as potential background EC modifiers for the purposes of this study. We then compared the COH trends in NJ and CA with the background EC data available from the late 1980s assessment of the industry, and the NJ data were much more strongly aligned with these earlier data than CA. For example, the Zaebst et al. (1991) assessment reported background EC geometric means that were approximately 2.2 times higher than those observed during the more recent TrIPS assessment (Smith et al. 2006), which corresponded to the temporal pattern of COH in NJ (2.2 times higher between base year 2000 with the 1988-89 period). Based on the fact that the NJ COH trend provided such a strong fit, with the additional benefit that it was available over the longest time period, we used the NJ COH data to adjust for background trends in this study.

Figure 3: Comparison of Background Trends 1985-2000

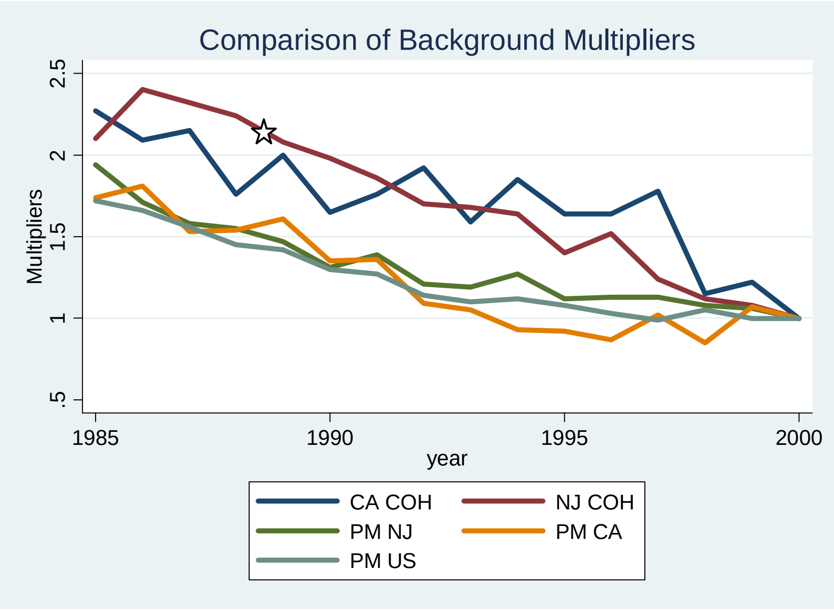

☆ Represents background levels detected during the earlier exposure assessment, which exactly correspond with the COH multiplier developed specifically from the NJ dataset.

References

Fraser MP, Buzcu B, Yue ZQ, McGaughey GR, Desai NR, Allen DT, Seila RL, Lonneman WA, Harley RA.2003. Separation of fine particulate matter emitted from gasoline and diesel vehicles using chemical mass balancing techniques. Environ Sci Technol 37:3904-9.

Hart JE, Yanosky JD, Puett RC, Ryan L, Dockery DW, Smith TJ, Garshick E, Laden F. 2009. Spatial modeling of PM10 and NO2 in the contintental United States, 1985-2000. Env Health Perspect 117:1690-1696.

Kleeman MJ, Schauer JJ, Cass GR. 2000. Size and composition distribution of fine particulate matter emitted from motor vehicles. Environ Sci Technol 34:1132-1142.

Riddle SG, Robert MA, Jakober CA, Hannigan MP, Kleeman MJ. 2008. Size-resolved source apportionment of airborne particle mass in a roadside environment. Environ. Sci. Technol 42:6580–6586.

Schauer JJ. 2003. Evaluation of elemental carbon as a marker for diesel particulate matter. J Exp Sci Environ Epidemiol 13:443–453.

Sheesley RJ, Schauer JJ, Garshick E, Laden F, Smith TJ, Blizcharz AP, DeMinter JT. 2009. Tracking personal exposure to particulate diesel exhaust in a diesel freight terminal using organic tracer analysis, J Exp Sci Environ Epidemiol 19:172-86.

Sheesley RJ, Schauer JJ, Smith TJ Garshick E, Laden F, Marr L, Molina L. 2008. Assessment of diesel particulate matter in the workplace: freight terminals. J Environ Monitor 10:305-14.

Smith TJ, Davis ME, Reaser P, Hart JE, Laden F, Heff A, Garshick E. 2006. Overview of particulate exposures in the US trucking industry. J Environ Monit 8:711-720.

Zaebst DD, Clapp DE, Blade LM, Marlow DA, Steenland K, Hornung RW, Scheutzle D, Butler J. 1991. Quantitative determination of trucking industry workers' exposures to diesel exhaust particles. Am Ind Hyg Assoc J 52:529–541.
